# Specimen Collection for Translational Studies in Hidradenitis Suppurativa

**DOI:** 10.1038/s41598-019-48226-w

**Published:** 2019-08-21

**Authors:** A. S. Byrd, Y. Dina, U. J. Okoh, Q. Q. Quartey, C. Carmona-Rivera, D. W. Williams, M. L. Kerns, R. J. Miller, L. Petukhova, H. B. Naik, L. A. Barnes, W. D. Shipman, J. A. Caffrey, J. M. Sacks, S. M. Milner, O. Aliu, K. P. Broderick, D. Kim, H. Liu, C. A. Dillen, R. Ahn, J. W. Frew, M. J. Kaplan, S. Kang, L. A. Garza, L. S. Miller, A. Alavi, M. A. Lowes, G. A. Okoye

**Affiliations:** 1 Department of Dermatology, Johns Hopkins University School of Medicine, Baltimore, MD 21231 USA; 2 Meharry Medical College, Nashville, TN 37208 USA; 3 University of Maryland School of Medicine, Baltimore, MD 21201 USA; 4 Systemic Autoimmunity Branch, National Institute of Arthritis and Musculoskeletal and Skin Diseases, National Institutes of Health, Bethesda, MD 20892 USA; 5 Department of Molecular and Comparative Pathobiology, Johns Hopkins University School of Medicine, Baltimore, MD 21205 USA; 6 Division of Clinical Pharmacology, Department of Medicine, Johns Hopkins University School of Medicine, Baltimore, MD 21205 USA; 7 Departments of Dermatology and Epidemiology, Columbia University, New York, NY 10032 USA; 8 Program for Clinical Research, Department of Dermatology, University of California San Francisco, San Francisco, CA 94143-0808 USA; 9 Stanford University School of Medicine, Stanford, CA 94305 USA; 10 Weill Cornell/Rockefeller/Sloan-Kettering Tri-Institutional MD-PhD Program, New York, NY 10065 USA; 11 Department of Plastic and Reconstructive Surgery, Johns Hopkins University School of Medicine, Baltimore, MD 21231 USA; 12 Department of Microbiology, Immunology, and Molecular Genetics, University of California Los Angeles, Los Angeles, CA 90095 USA; 13 Department of Dermatology, Liverpool Hospital, Sydney, NSW 2170 Australia; 14 Ingham Institute of Applied Medical Research, Liverpool, Sydney, NSW 2170 Australia; 15 University of New South Wales, Sydney, NSW 2033 Australia; 16 Department of Medicine (Dermatology), University of Toronto, Toronto, Ontario M1C 1A4 Canada; 17 Division of Dermatology, Women’s College Hospital, Toronto, ON M5S 1B2 Canada; 18 The Rockefeller University, New York, NY 10065 USA; 19 Present Address: Department of Dermatology, Howard University College of Medicine, Washington, DC 20060 USA

**Keywords:** Chronic inflammation, Chronic inflammation, Autoinflammatory syndrome, Autoinflammatory syndrome

## Abstract

Hidradenitis suppurativa (HS) is a chronic inflammatory disorder characterized by painful nodules, sinus tracts, and scars occurring predominantly in intertriginous regions. The prevalence of HS is currently 0.053–4%, with a predominance in African-American women and has been linked to low socioeconomic status. The majority of the reported literature is  retrospective, population based, epidemiologic studies. In this regard, there is a need to establish a repository of biospecimens, which represent appropriate gender and racial demographics amongst HS patients. These efforts will diminish knowledge gaps in understanding the disease pathophysiology. Hence, we sought to outline a step-by-step protocol detailing how we established our HS biobank to facilitate the formation of other HS tissue banks. Equipping researchers with carefully detailed processes for collection of HS specimens would accelerate the accumulation of well-organized human biological material. Over time, the scientific community will have access to a broad range of HS tissue biospecimens, ultimately leading to more rigorous basic and translational research. Moreover, an improved understanding of the pathophysiology is necessary for the discovery of novel therapies for this debilitating disease. We aim to provide high impact translational research methodology for cutaneous biology research and foster multidisciplinary collaboration and advancement of our understanding of cutaneous diseases.

## Introduction

Hidradenitis suppurativa (HS) is a chronic inflammatory disorder characterized by painful nodules, sinus tracts and scars that occur predominantly in intertriginous regions^1,2^. The wide range of reported prevalence is 0.05–4%, but may be an underestimation since the diagnosis is often delayed or missed altogether^1–3^. HS occurs more frequently in women (female to male ratio of 3:1) and patients of African descent^1,4–6^. Patients suffering from HS consistently report an impaired quality of life and possess among the poorest scores reported when using the dermatology quality of life index (DLQI)^7,8^. The underlying etiology of HS is presently unknown and current therapy is often inadequate or ineffective^1–3,9^. Consequently, there is a need for better understanding of the pathogenesis of HS and development of novel therapies.

To this end, we propose a HS-specific biobanking protocol to establish a standardized process for consistent and ethical data collection and analysis for future clinical and basic science studies. Biobanks are well-organized collections of human biological material which are stored for research and can help drive translational research^10–12^. However, differences in biospecimen collection and management amongst biorepositories and institutions in this age of national and international collaboration can lead to variability in quality and molecular integrity that ultimately interferes with results and reproducibility^13,14^. Though there are existing biobanking protocols for many other conditions^15,16^, there is no central source to describe best practices for ethical issues, biospecimen collection, processing techniques and biorepository development (storage) for HS biobank development. Biobanking is particularly important for complex and largely understudied diseases like HS^10,12^. This protocol was successfully instituted at the Johns Hopkins University School of Medicine (JHUSOM) and has already contributed to translational studies^17^.

**Figure 1:**
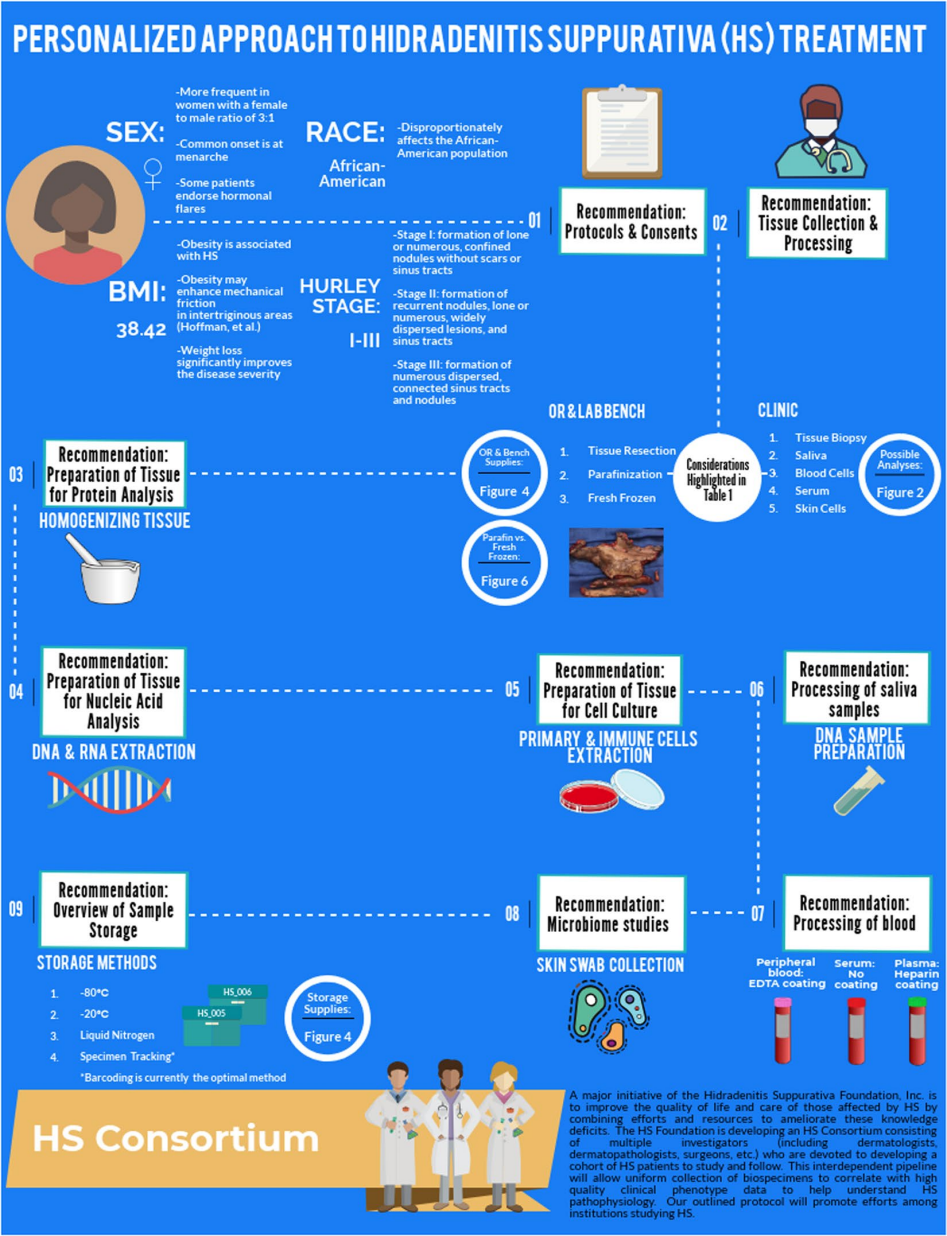
Overview of the personalized approach taken to biobank HS samples and the goals of the HS Consortium. Figure produced with Piktochart.

The purpose of this manuscript is to present a detailed, step-by-step protocol for collecting HS specimens and building an HS biobank. During the collection of biospecimens, careful clinical phenotyping, patient histories and in selected cases, ultrasound imaging, should be accompanied by photography. Figure 1 provides an overview to our approach to specimen collection for translational studies in HS, emphasizing the role of collecting clinical phenotypic data for correlation with basic laboratory findings. There are a number of unique challenges to collecting biospecimens for translational research in HS. Herein, we acknowledge and present our approaches to address them.

## Methods

All samples are collected, stored and used in agreement with the ethical and research guidelines set by biobanking privacy laws^18^ and the Institutional Review Boards (IRB) of JHUSOM and University of California, San Francisco. The methods for specimen collection for genetic studies were approved at Columbia University Irving Medical Center. Informed consent was obtained from all participants and/or their legal guardian(s). These methods can be applied elsewhere after institutional IRB approval, further evolving new technologies and knowledge.

## Overview of Biospecimen Collection

### Biospecimen categories and applications- detailed in Figs 2 and 3

i. Skin biopsies and surgically resected tissue samples can be collected from diseased tissue (“lesional”), areas adjacent to the diseased tissue but without gross evidence of disease (“peri-lesional”), normal-appearing tissue of an HS patient (“unaffected”), as well as healthy individuals (“normal”). Precise descriptions of the morphology, disease severity and exact location of lesions biopsied enable experimental comparisons of morphologically similar lesions from different individuals. Where possible, the lesional, peri-lesional and unaffected skin should be collected from the same anatomical region, as there are region-specific differences in epidermal appendages and the cutaneous microbiome^19^. Photographic documentation before and after sample collection can aid in future clinical-pathological correlations. Punch biopsies (4 mm or larger in size) and excisional biopsies should be deep enough to include subcutaneous fat. Surgically resected tissue tends to provide larger and deeper tissue sections than biopsies, providing opportunities for more assays and increasing the tissue repository for future evaluation. Tissue sections can be utilized for histology, immunohistochemistry (IHC), and immunofluorescence (IF). Tissue proteins can be obtained for Western Blotting (WB), Enzyme-linked immunosorbent assay (ELISA), zymography and tissue proteomics^20^. Whole tissues can be processed for keratinocytes, fibroblasts and immune cells. Nucleic acids can also be extracted for transcriptomic, genetic analysis and microbiome analysis^21–23^.

**Figure 2:**
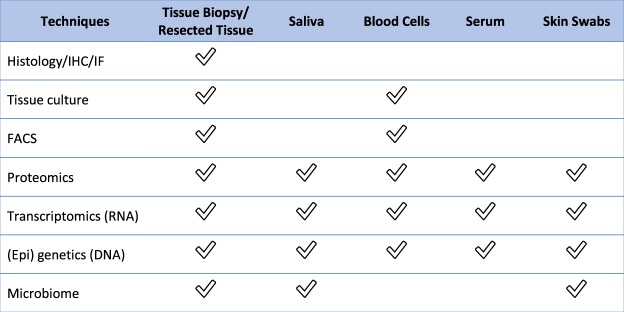
Analysis techniques possible for collected biospecimens. Checks denote whether each analysis technique can be performed with a collected biospecimen.

**Figure 3:**
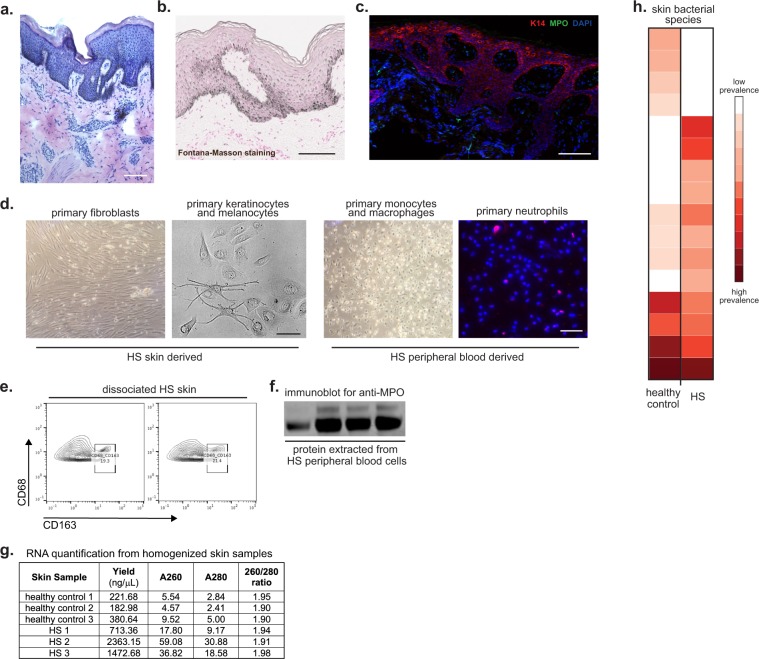
Examples of analysis techniques for collected biospecimens. (**a**) Hematoxylin & Eosin staining of HS lesional skin from fresh-frozen tissue. **(b)** Fontana-Masson staining of HS skin from paraffin-embedded tissue. Scale bar: 100 μm. **(c)** Immunofluorescence of HS skin for keratin 14 (red), MPO-myeloperoxidase (green), and DAPI (blue). **(d)** HS skin was digested and primary fibroblasts (*first panel*) and primary epidermal cells- keratinocytes and melanocytes- (*second panel*) were cultured. Peripheral blood was collected from HS patients and monocytes/macrophages (*third panel*) and neutrophils (*fourth panel*) were isolated and cultured. Scale bar: 50 μm. **(e)** HS skin was digested into single cell suspension and stained for flow cytometry analysis. **(f)** Protein was extracted from peripheral blood cells and used to perform Western blot. Uncropped Western Blot in Supplemental Fig. S1. **(g)** RNA was extracted from homogenized healthy control and HS skin and was quantified with a spectrophotometer. The 260/280 absorbance ratio of ~2 represents the purity and quality of the RNA. **(h)** Heatmap representing the microbiome of healthy control and HS skin developed by next-generation sequencing.
ii. Saliva can yield high quality human RNA/DNA to assess biomarkers or genetic mutations and polymorphisms in HS patients. Direct^24–26^ or oral swab^27^ collection of saliva may also be utilized to understand changes in the oral microbiome that may be linked to systemic disease manifestations. Bacterial RNA/DNA extraction methods have minimal if any effect on saliva microbiome profiles, making saliva an ideal biospecimen for this analysis^28^. In addition, saliva can be used for proteomic analysis^29,30^.
iii. Blood cells, such as peripheral blood mononuclear cells (PBMCs) and neutrophils, can be isolated to provide insight into the systemic response and contributions to HS^31,32^. These cells may be used for flow cytometry^17,32–34^, proteomics^35^, transcriptomic and genetic analysis as well as functional analyses^36,37^, including cell culture/co-culture experiments^38,39^.
iv. Serum can also aid in understanding the systemic effects of HS. Inflammatory and other biomarkers may be identified using either large scale proteomic platforms or individual analyses, and can be related to onset, specific disease stages, and/or disease progression^40^. Likewise, RNA and DNA can also be extracted for transcriptomic and genetic analysis, respectively^22,41^.
v. Skin swabs can be used to catalog the composition of and shifts in the HS skin microbiome. Skin cells, chemicals and microbes collected on skin swabs can be used to analyze molecular and microbial characteristics via mass spectrometry and microbial 16 S rRNA amplicon sequences^42^, as well as high-throughput next-generation sequencing^23,43,44^. Details have been outlined in Figs. 2 and 3.

### Outpatient versus inpatient biospecimen collection

**Figure 4:**
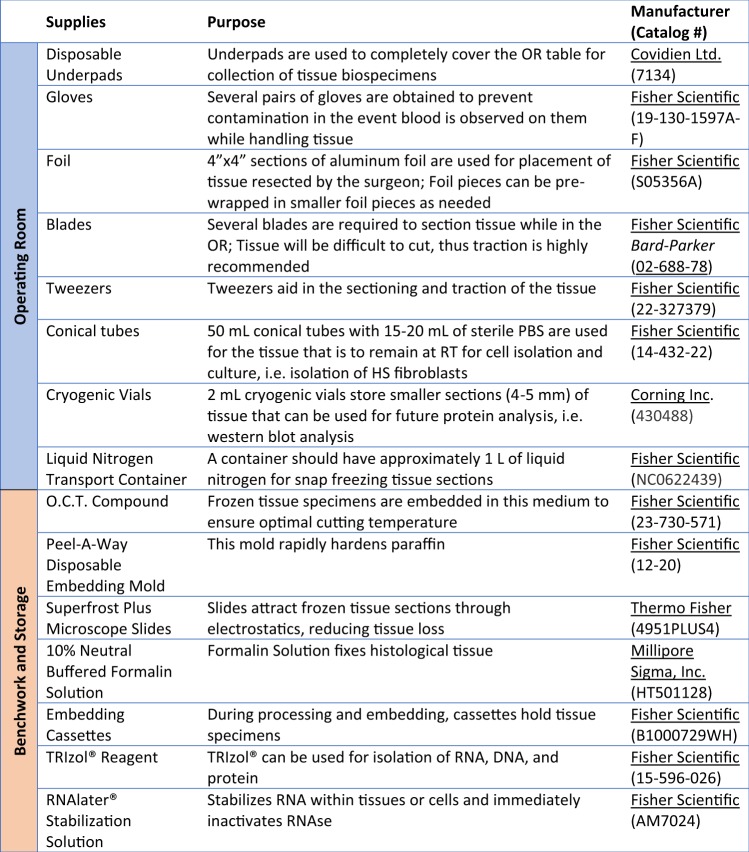
Supplies for operating room, benchwork, and storage. Listed are the supplies necessary for the operating room, benchwork, and storage when working with HS biospecimens as well as their purpose and manufacturers.

Biospecimens may be collected during outpatient clinic visits or in the operating room (OR). The advantages and disadvantages of each mode of collection have been outlined in Table 1. Biospecimens collected during outpatient visits allows for concurrent clinical correlations. Collection of HS tissue resections in the OR provides abundant tissue for analysis; however, the clinical context may not be fully assessed if the patient was not evaluated clinically by the investigators prior to surgery. When collecting surgically resected tissue in the OR, it is important to bring specific supplies to the site of tissue collection (Fig. 4).

**Table 1:** Comparative analysis of clinical outpatient vs. operating room tissue collection.

Considerations	Outpatient Clinic	OR/Surgical Resection
Patient report	**(+)** More relaxed atmosphere; Patients are more willing to listen to ongoing research efforts, ask questions, etc.	**(−)** Patients are usually anxious about surgery and less willing to engage in conversation/ questions asked about the disease
Collection of tissue samples	**(+)** Patients will be more willing to provide smaller tissue sections for research biospecimens **(+)** Allows for biopsies of lesional, perilesional, and unaffected tissue all from the same patient **(−)** Tissue section size will be limited/smaller (approx. 2-4 mm)	**(+)** Surgical removal of tissue is both therapeutic and allows for research biospecimens **(-)** More difficult to collect various tissue sections **(+)** Larger tissue sections
Healing time	**(+)** Smaller biopsies can be sutured for faster healing	**(−)** Open wounds require longer healing time and increase the risk of post-surgical infections
Collection of other biospecimens	**(+)** Very feasible to collect samples including saliva	**(−)** Usually not feasible, especially considering coordination needed with OR team.
Time	**(−)** If the clinic schedule is delayed, patients are less willing to participate	**(−)** The OR schedule can be delayed
Anatomy/Structure of lesions	**(−)** Only allows for surface examination of the lesions	**(+)** Allows full visualization of the anatomic location of lesions, including epithelization, sinus tracts, etc.
Cost	**(+)** Less expensive	**(−)** More expensive

## Recommendations for Tissue Collection- Outlined in Table 2

### Recommendation 1. Consents and protocols

Institution-specific IRB-approved protocols and consents provide the opportunity for prospective biobanking and tissue analysis, retrospective analysis of archived tissue and multi-institutional collaborations. During clinical encounters, patients should be informed about ongoing HS research studies and offered the opportunity to participate. If interested, informed consent should be obtained, allowing for biospecimen collection, analysis of collected material, permission to contact for future studies and for clinical chart reviews. When tissue is collected in the OR, consent may be obtained immediately prior to surgery concurrent with informed consent for the surgical procedure. Alternatively, informed consent for the study can be obtained in advance of surgery. In the authors’ experience, the latter process allows the patient more time for questions and consideration of the implications of their participation.

**Table 2:** Recommendations for tissue and biospecimen collection.

Recommendations	Overview
Consents and Protocols	IRB approved consents and protocols Tissue and biospecimens are collected and utilized for treatment recommendations and research
Tissue collection & processing	Collect → Observe → Proper storage Transport from OR to bench Proper storage of lysate, RNA, OCT, and paraffin samples Cryostat sectioning of tissue for IHC and IF
Preparation of tissue for protein analysis	Homogenize whole tissue for Western Blot, ELISA, proteomics, etc.
Preparation of tissue for nucleic acid analysis	DNA/RNA extraction for genetic sequencing and analysis
Preparation of tissue for cell culture- Primary and immune cell extraction	Extraction of primary cells Cell culture of fibroblasts, keratinocytes, and melanocytes Extraction of immune cells
Processing of saliva samples	DNA sample preparation
Blood processing for serum, nucleic acid and immune cell isolation	Collection and separation, isolation, flow cytometry, RNA extraction
Skin swabs for microbiome studies	Sequencing and analysis for evaluation of microbial composition
Overview of sample storage	−20 °C, −80 °C, liquid nitrogen options for storage

### Recommendation 2. Tissue collection and processing

**Figure 5:**
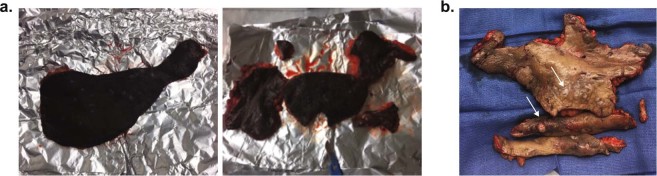
(**a**) A large piece of freshly resected HS tissue on foil (*left*). The tissue can be further sectioned using a scalpel to enable proper storage and further studies (*right*). **(b)** Arrows indicate areas of apparent nodules and lesions on freshly resected HS tissue.

*OR:* Tissue should be placed on prepared aluminum foil pieces once resected in the OR. After gross appearance of the tissue is observed and recorded, including photographs if possible (Fig. 5), the tissue should be partitioned as follows:

- Five 0.3–0.5 cm sections should be placed into individual 2 mL cryogenic vials.
- Three 0.5–1 cm sections should be wrapped in foil for optimal cutting tissue compound (OCT) embedding, paraffin and RNA extraction. Timely processing prevents RNA degradation.
- One larger tissue section (approximately 2.5 cm) should be placed in a 50 mL conical tube with sterile Phosphate-buffered saline (PBS), kept at room temperature and either processed or placed on ice within 30 minutes. If isolating immune cells, this tissue section must be processed on the same day it is collected.
- Remaining sections should be wrapped in foil to be stored for other analyses.

All cryogenic tubes containing tissue sections and foil wrapped tissue sections should be placed in a container with liquid nitrogen for transport back to the lab. If immediate snap freezing is not feasible, tissues should be frozen within 30 minutes of resection. Each patient should have a representative tissue section placed in formalin and sent for routine histopathologic examination and archiving by the surgical team.

#### Outpatient clinic

The biospecimen noted above can be collected during outpatient visits with appropriate staffing and supplies. Smaller tissue samples can be obtained using punch biopsies or small elliptical excisions and can be treated in the aforementioned manner. Table 1 outlines helpful considerations when comparing biospecimen collection in outpatient or OR settings

*Initial tissue processing:* The specimens should be quickly returned to the laboratory bench, paying special attention to the  liquid nitrogen level in order to prevent spills. The bench should be lined with disposable underpads. The larger tissue sections and the individual cryogenic vials should be placed in a storage box with a de-identified label (e.g. HS_001) and stored at −80 °C for future use.

**Figure 6:**
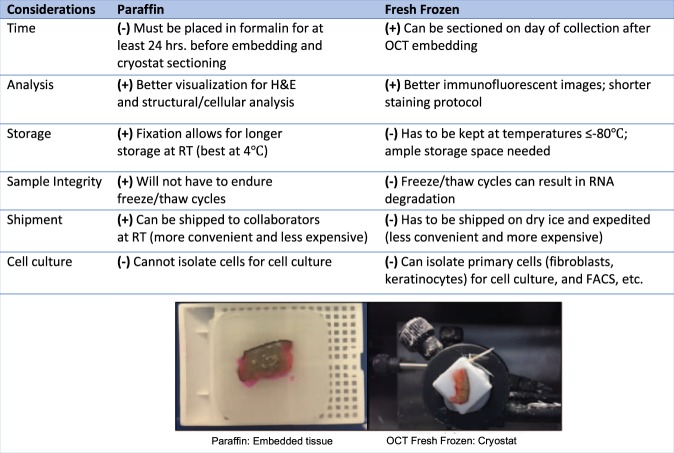
Comparative analysis of paraffin and fresh frozen tissue with images. Positive and/or negative aspects for each consideration when processing tissue as paraffin or fresh frozen are listed. Images of possible analysis techniques are shown. (+) denotes positive aspect; (−) denotes negative aspect.

Samples can be processed as fresh frozen or as formalin fixed paraffin embedded tissue (FFPE). Both incur advantages and disadvantages, outlined in Fig. 6. For the tissue designated for OCT (fresh frozen tissue), ‘peel-away’, disposable and variously sized histology cryomolds can be used. The cryomold should be slowly filled with enough OCT media to cover the tissue section, paying special attention to avoid bubbles. Forceps should be used to place the tissue section in an orientation such that the epidermis and dermis can be visualized on a cross-sectional cut (Fig. 6). The cryomold should be immersed, not fully, into liquid nitrogen (or slurry of methanol and dry ice) for 30 seconds or until nearly frozen. At this point, it can be transferred to a foam cooler of dry ice. Individual samples can be wrapped in labeled foil and sealed in a plastic bag. These samples can be stored at −80 °C or sectioned with a cryostat immediately. If continuing with the cryostat sectioning, samples may be cut into 3–8 μm sections and placed on labeled plus-plus glass slides. Cryostat sectioned slides should immediately be placed at −20 °C or −80 °C for permanent storage.

Tissue designated for FFPE should be placed in labeled cassettes and submerged in 10% formalin. The container should be stored at 4 °C for at least 24 hours for proper fixation before paraffin embedding.

Tissue designated for RNA should be placed in a 50 mL conical tube with 10–15 mL of TRIzol® or RNA*later®*, and immediately stored at −80 °C or future use. Figure 4 outlines specific supplies and resources.

### Recommendation 3. Preparation of tissue for protein analysis

#### Homogenization of whole tissue

Designated tissue sections can be homogenized and utilized to assess proteins, using experiments such as WB, ELISA and proteomics. Several 3–5 mm tissue sections should be placed in a mortar with enough liquid nitrogen to flash freeze the tissue. Using a pestle, the flash frozen tissue should be ground into powder-like substance to which lysis buffer should be added and processed accordingly^45^. The lysate can be stored at −80 °C until further use^45^.

### Recommendation 4. Preparation of tissue for nucleic acid analysis

#### DNA and RNA extraction

The recommended protocol above yields a frozen specimen from which you can extract nucleic acids using purification protocols or manufactured kits^43,46–52^. The specimen can be stored at −80 °C until required.

### Recommendation 5. Preparation of tissue for cell culture

#### Extraction of primary cells from tissue

Fresh tissue placed in sterile PBS can be used to isolate primary cells. Tissue samples can be digested using various methods^53–57^ to derive single cell suspensions. This allows for the isolation of cell types of interest via subsequent extracellular and/or intracellular staining and flow cytometry.

For fibroblast and keratinocyte isolation specifically, tissue can be separated into the dermis (fibroblasts) and epidermis (keratinocytes and melanocytes) as previously described^58^. Confluent cultured HS fibroblasts, keratinocytes, and melanocytes are pictured in Fig. 3d. Primary cultures of human fibroblasts and keratinocytes can also be used to create induced pluripotent stem cells (iPSC), which provide a pathway to model and treat human diseases^59–61^.

#### Extraction of immune cells from tissue

As described in the previous section, skin tissue samples can be digested to derive single cell suspensions, which can be stained and sorted using flow cytometry for specific immune cell selection^53–57^. Specific to T cells, skin tissue samples can be minced accordingly, and subsequently cultured. Alternatively, explants can be cultured directly in 24-well plate wells with a small decrease in number of T cells isolated^62^. We encourage our readers to consider that recent studies suggest HS is a complex immune-mediated process and this method that focuses on a single immune cell type, the T cell, is limited in scope.

### Recommendation 6. Processing of saliva samples

#### DNA sample preparation

Saliva can be self-collected in specialized tubes that allow for ambient storage for up to five years. DNA extraction reagents and protocols are provided by kit manufacturers. Extracted DNA can be stored at −20 °C (−80 °C for storage beyond 6 months) in aliquots. Each kit typically yields 100 μg of DNA, ensuring adequate amounts for multiple genetic analyses^63^. Saliva can also be used for proteomic^29^, transcriptomic^64^ and microbiome analysis^28,65^.

### Recommendation 7. Blood processing for plasma, serum, nucleic acid and immune cell isolation

#### Peripheral blood collection and separation

Specific tubes which can be used are indicated in Fig. 1. To evaluate peripheral blood soluble mediators, plasma and serum can be separated from the blood by centrifugation^33^, removed as a clear layer, divided into aliquots (minimizing freeze thaw cycles) and stored at −80 °C for subsequent analyses. During serum collection, clotted blood will be discarded after centrifugation. Although serum provides a higher sensitivity for biomarker detection, experimental question and design determine the use of serum and/or plasma^66–68^.

#### Isolation of immune cells

Following plasma isolation, remaining blood is separated by density gradient centrifugation to collect PBMCs^17,69,70^. Cells not being analyzed immediately can be frozen in aliquots. Polymorphonuclear cells (PMN; neutrophils), can also be isolated in a similar manner^33^,but must be used within 5–10 hours after isolation due to short lifespan.

#### Flow cytometry preparation

If possible, a portion of the PBMC can be immunostained for extracellular and intracellular flow cytometric analyses^71^.

#### Selection of desired cell preparations

Immunomagnetic separation to isolate specific cell subpopulations can also be performed from PBMCs. Positive or negative selection may be performed to obtain desired cell populations, depending on the investigator’s preference^72^.

#### RNA extraction

RNA can be isolated from PBMC or specific cell populations using TRIzol^®^. Alternatively, RNA may be isolated from whole blood by standardized kits (e.g. QIAamp RNA Blood Mini Kit, Qiagen, MD, USA). Upon isolation, RNA purity and concentration should be determined (Fig. 3g). RNA should be aliquoted for long term storage at −80 °C.

#### DNA extraction

Within a day of receiving the sample, peripheral blood collected into a 6 mL tube (with EDTA) can be shipped to the processing location at room temperature, within a 1-week window. DNA can be extracted from PBMCs using standardized kits (e.g. PUREGENE DNA isolation kit, Qiagen, MD, USA)^73^, and aliquoted for long term storage at −20 °C. However, ideally neutrophils should be isolated on the day of blood collection.

### Recommendation 8. Skin swab collections for microbiome studies

Study participants should prepare skin using an unscented, non-antibacterial soap for hygiene for 7 days; avoiding all bathing or washing 24 hours prior to sampling, topical antiseptics for 7 days and systemic antibiotics 6 months prior to sampling. Samples should be collected from selected body sites with no prior cleaning or preparation of the skin surface and site of swab should be documented. Swabs should be obtained from a 4 cm^2^ area using cotton-tipped applicators soaked in enzymatic lysis buffer (20 mM Tris pH 8, 2 mM EDTA, and 1.2% Triton X-100). Negative controls of ambient air swabs should be collected after each sampling. All samples should be stored at −80 °C until further processing^74^. Skin swabs can also be used for transcriptomic^74^ and epigenetic^75^ analysis.

### Recommendation 9. Overview of sample storage and barcoding^76^ – Fig. 1

## Limitations and Discussion

Recommendations have been outlined in Fig. 1 and Table 2.  One important limitation to note is that variation in important inflammatory cytokines can be associated with the methods of tissue collection, time to freezing, patient age, the amount of cutaneous ultraviolet light exposure around the time of tissue collection, as well as the biospecimen storage duration^77–79^. Whilst validation studies provide some reassurance to the stability of serum and saliva samples^25,78^, the existing literature on cutaneous biorepository samples is less robust^77^. Hence, any case control matching would require thorough demographic matching to ensure the most accurate and reliable results from future studies. Obstacles may be encountered during the development of an HS biobank and we have outlined some common troubleshooting tips in Supplementary Table S1.

Many major gaps in our understanding of HS exist^80^. The majority of the reported literature are retrospective, population based, epidemiologic studies. The published basic science research reports are usually studies with small sample sizes and with limited clinicopathological correlations. Certainly, the lack of an animal model limits a deeper understanding of cellular and pathologic mechanisms^81,82^. Therefore, there is a need to establish a translational approach to studying this devastating disease.

The availability of biobanked specimens from clinically well-characterized HS patients with appropriate gender and racial representation will help address these knowledge gaps. When collecting biospecimens, a thorough clinical assessment is needed to improve our understanding of the correlations between the clinical phenotype and the generated molecular data. Thus, detailed descriptions of phenotypes, exposures and treatment outcomes should be included in the clinical evaluation of these patients. Additionally, standardization of disease severity measures and nomenclature are imperative for the generalizability of data collected.

The public’s use of the Internet for obtaining and exchanging health-related information has created opportunities to rapidly engage patients and efficiently collect data through Internet surveys. Additionally, the standardization of electronic health records and changes in policy that give patients access to their own records also provides an opportunity to receive patient-supplied clinical records to complement survey data.

Biobanking serves as a major driver of translational research. Although there are multiple methods for performing various scientific analyses, we are proposing a general roadmap and preferable methods for those conducting HS research and studying clinical manifestations in order to render reproducible and reliable results and conclusions. It is our hope that this protocol will promote the development of other institution-specific biobanks for laboratory studies and collaborative efforts to improve our understanding of HS. These protocols can be updated as new technologies drive advanced studies.

## Supplementary information

  Supplementary Figure S1: Uncropped Western Blots, Supplementary Table S1: Common Troubleshooting Tips in Biospecimen Collection
