# Supplementary material for: Specimen Collection for Translational Studies in Hidradenitis Suppurativa
Source: Sci Rep. 2019 Aug 21;9:12207. doi: 10.1038/s41598-019-48226-w (PMC6704132; doi:10.1038/s41598-019-48226-w)
Supplement: Supplementary file 1 — Supplementary Figure S1: Uncropped Western Blots, Supplementary Table S1: Common Troubleshooting Tips in Biospecimen Collection [file 41598_2019_48226_MOESM1_ESM.docx]

Supplementary Information

Specimen Collection for Translational Studies in Hidradenitis Suppurativa

A.S. Byrd, Y. Dina, U.J. Okoh, Q.Q. Quartey, C. Carmona-Rivera, D.W. Williams, M.L. Kerns, R.J. Miller, L. Petukhova, H.B. Naik, L.A. Barnes, W.D. Shipman, J.A. Caffrey, J.M. Sacks, S.M. Milner, O. Aliu, K.P. Broderick, D. Kim, H. Liu, C.A. Dillen, R. Ahn, J.W. Frew, M.J. Kaplan, S. Kang, L.A. Garza, L.S. Miller, A. Alavi, M.A. Lowes, G.A. Okoye

Supplementary Figure S1: Uncropped Western Blots

Supplementary Table S1: Common Troubleshooting Tips in Biospecimen Collection

from Figure 3f: anti-MPO


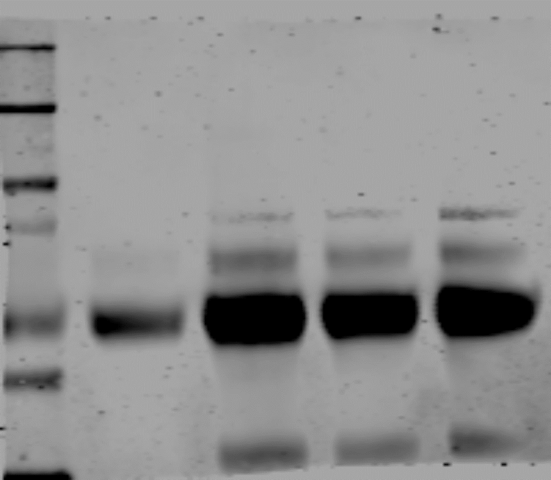


**Supplementary Figure S1: Uncropped Western Blots**. Western Blot from Figure 3f. Red box indicates the portion of the blot that was used in the figure.

Supplemental Table S1: Common Troubleshooting Tips in Biospecimen Collection

| **Potential Problems** | **Possible Reasons** | **Possible Solutions** |
| --- | --- | --- |
| Small number of patient samples | Difficulty identifying patients | 1. Inform colleagues with department or other departments at institution (e.g. general surgery, plastic surgery, dermatology). 2. Collaborate with colleagues at different institutions. 3. Community outreach to recruit patients. |
|  | Difficulty with obtaining consent | 1. Employ support staff to focus on patient enrollment. 2. Use easy-to-read patient-friendly information to explain studies to patient. |
| Poor sample quality | Poor workflow | 1. Ensure surgeons are aware of tissue collection, OR is prepared for collection, and that reagents in lab are ready for tissue processing. 2. Ensure there is enough personnel present to efficiently and quickly transfer tissue from OR to the laboratory and begin processing. 3. Plan use of tissue beforehand and decide what assays are possible with amount of tissue anticipated. |
|  | Infrastructure issues | 1. Make sure laboratory space is within appropriate distance to OR or that appropriate conditions are met to transport tissue on ice in a timely manner. 2. Ensure appropriate storage of tissue and specimens (e.g. storage at appropriate temperature and optimal functioning of 4°C, -20°C, and -80°C units). |
|  | Inappropriate Technique | 1. Ensure appropriate reagents, kits, and techniques are being used to process human samples. 2. Various assays with biospecimens may need to be optimized specifically for the user’s equipment and reagents, which may take quite some time. |
| Difficulty with obtaining interest in or use of biobank | Decreased scientific productivity of biobank | 1. Use biobank efficiently to publish high-quality manuscripts in both basic science and clinical journals. 2. Ensure colleagues using biobank have appropriate plan to publish findings from biobank. |
|  | Poor public awareness of biobank | 1. Present biobank or associated data at conferences, seminars, and symposiums for colleagues to become aware of its existence and potential use. 2. Ensure that biobank users or collaborators always acknowledge the biobank in all manuscripts or public presentations. |
